# Supplementary material for: Assessment of Vibrio parahaemolyticus levels in oysters (Crassostrea virginica) and seawater in Delaware Bay in relation to environmental conditions and the prevalence of molecular markers to identify pathogenic Vibrio parahaemolyticus strains
Source: PLoS One. 2020 Dec 3;15(12):e0242229. doi: 10.1371/journal.pone.0242229 (PMC7714158; doi:10.1371/journal.pone.0242229)

PLOS ONE

Analysis of Molecular Markers in the Environmental Strains of Vibrio Parahaemolyticus Isolated from the Oyster (Crassostrea virginica) and Water Samples of Delaware Bay

--Manuscript Draft--

**Manuscript Number:**	PONE-D-20-19028
**Article Type:**	Research Article
**Full Title:**	Analysis of Molecular Markers in the Environmental Strains of Vibrio Parahaemolyticus Isolated from the Oyster (Crassostrea virginica) and Water Samples of Delaware Bay
**Short Title:**	Analysis of Vibrio Parahaemolyticus in Delaware Bay
**Corresponding Author:**	Gulnihal Ozbay<br>Delaware State University<br>Dover, Delaware UNITED STATES
**Keywords:**	Vibrio parahaemolyticus; Vibrio parahaemolyticus metalloprotease (vpm); thermolabile direct hemolysin (tlh); thermostable direct hemolysin (tdh); TDH-related hemolysin (trh); transmembrane DNA-binding protein (toxR); oysters; Delaware Bay; Mid-Atlantic
**Abstract:**	This study is aimed to identify Vibrio parahaemolyticus in oyster and seawater samples collected from Delaware Bay from June through October of 2016. CHROMagar Vibrio medium was used to identify V. parahaemolyticus , in which presumptive V. parahaemolyticus colonies display mauve color. The highest mean levels of V. parahaemolyticus were 9.63×10 3 CFU/g and 1.85×10 3 CFU/mL in the oyster and seawater samples during the month of July. The lowest detectable limit of V. parahaemolyticus in oyster and seawater samples was 1.7×10 CFU/g (or mL) during the months of September and October. We have confirmed that V. parahaemolyticus levels were positively correlated with temperature in oyster and seawater samples. V. parahaemolyticus isolates (n=165) were further characterized by screening the species-specific thermolabile hemolysin (tlh) gene, the pathogenic thermostable direct hemolysin (tdh)/ thermostable related hemolysin (trh) genes, the regulatory transmembrane DNA-binding gene (toxR), and V. parahaemolyticus metalloprotease (vpm) gene. Of the 165 isolates, 137 (83%), 110 (66.7%), and 108 (65%) were tlh + , vpm + , and toxR + , respectively. Among the V. parahaemolyticus (tlh +) isolates, 7 (5.1%) and 15 (10.9%) were tdh + and trh + , respectively, and 24 (17.5%), only oyster isolates, were positive for both genes. Potential pathogenic strains that possessed tdh and/or trh were notably higher in oyster (39%) than seawater (15.6%) isolates. Occurrence of total V. parahaemolyticus (tlh +) were not necessarily proportional to the potential pathogenic V. parahaemolyticus . Co-occurrence of the five genetic markers were observed only among oyster isolates. Coexistence analysis of the gene markers revealed a relatedness potential of tdh occurrence with vpm . We believe exploring the role of V. parahaemolyticus metalloprotease and whether it involved in the toxic activity of the thermostable direct hemolysin (TDH) protein can be of significance. We expect our project outcomes will provide some foundation for future studies regarding pathogenic Vibrio dynamics in relation to environmental quality.
**Order of Authors:**	Esam Almuhaideb, M.D.
	Lathadevi K. Chintapenta
	Amanda Abbott
	Salina Parveen
	Gulnihal Ozbay
**Additional Information:**	
**Question**	**Response**
**Financial Disclosure**	This study was funded by:<br>1- United States Department of Agriculture-National Institute of Food and Agriculture-

Powered by Editorial Manager® and ProduXion Manager® from Aries Systems Corporation

Enter a financial disclosure statement that describes the sources of funding for the work included in this submission. Review the submission guidelines for detailed requirements. View published research articles from *PLOS ONE* for specific examples.<br><br>This statement is required for submission and **will appear in the published article** if the submission is accepted. Please make sure it is accurate.<br><br>**Unfunded studies**<br>Enter: *The author(s) received no specific funding for this work.*<br><br>**Funded studies**<br>Enter a statement with the following details:<br>• Initials of the authors who received each award<br>• Grant numbers awarded to each author<br>• The full name of each funder<br>• URL of each funder website<br>• Did the sponsors or funders play any role in the study design, data collection and analysis, decision to publish, or preparation of the manuscript?<br>• **NO** - Include this sentence at the end of your statement: *The funders had no role in study design, data collection and analysis, decision to publish, or preparation of the manuscript.*<br>• **YES** - Specify the role(s) played.<br><br>* typeset	Capacity Building Grant for Vibrio Project (2014-38821-22430) - funded AA, GO, and SP.<br>https://nifa.usda.gov/<br><br>2- United States Department of Agriculture-Evans-Allen Grant (DELXDSUGO2015 Award) - funded KC.<br>https://nifa.usda.gov/program/agricultural-research-1890-land-grant-institutions<br><br>The funders had no role in study design, data collection and analysis, decision to publish, or preparation of the manuscript.
**Competing Interests**<br><br>Use the instructions below to enter a competing interest statement for this submission. On behalf of all authors, disclose any competing interests that could be perceived to bias this work—acknowledging all financial support and any other relevant financial or non-financial competing interests.<br><br>This statement **will appear in the published article** if the submission is accepted. Please make sure it is accurate. View published research articles from *PLOS ONE* for specific examples.	The authors have declared that no competing interests exist.

Powered by Editorial Manager® and ProduXion Manager® from Aries Systems Corporation

**NO authors have competing interests**<br><br>Enter: *The authors have declared that no competing interests exist.*<br><br>**Authors with competing interests**<br><br>Enter competing interest details beginning with this statement:<br><br>*I have read the journal's policy and the authors of this manuscript have the following competing interests: [insert competing interests here]*<br><br>* typeset	
**Ethics Statement**<br><br>Enter an ethics statement for this submission. This statement is required if the study involved:<br><br>• Human participants<br>• Human specimens or tissue<br>• Vertebrate animals or cephalopods<br>• Vertebrate embryos or tissues<br>• Field research<br><br>Write "N/A" if the submission does not require an ethics statement.<br><br>General guidance is provided below. Consult the submission guidelines for detailed instructions. **Make sure that all information entered here is included in the Methods section of the manuscript.**	Field sampling collection was granted by the Department of Natural Resources and Environmental Control (DNREC 2015 & 2016).<br>Delaware State University, Department of Agriculture and Natural Resources

Powered by Editorial Manager® and ProduXion Manager® from Aries Systems Corporation

**Format for specific study types**<br><br>**Human Subject Research (involving human participants and/or tissue)**<br>• Give the name of the institutional review board or ethics committee that approved the study<br>• Include the approval number and/or a statement indicating approval of this research<br>• Indicate the form of consent obtained (written/oral) or the reason that consent was not obtained (e.g. the data were analyzed anonymously)<br><br>**Animal Research (involving vertebrate animals, embryos or tissues)**<br>• Provide the name of the Institutional Animal Care and Use Committee (IACUC) or other relevant ethics board that reviewed the study protocol, and indicate whether they approved this research or granted a formal waiver of ethical approval<br>• Include an approval number if one was obtained<br>• If the study involved *non-human primates*, add *additional details* about animal welfare and steps taken to ameliorate suffering<br>• If anesthesia, euthanasia, or any kind of animal sacrifice is part of the study, include briefly which substances and/or methods were applied<br><br>**Field Research**<br><br>Include the following details if this study involves the collection of plant, animal, or other materials from a natural setting:<br>• Field permit number<br>• Name of the institution or relevant body that granted permission	
**Data Availability**<br><br>Authors are required to make all data underlying the findings described fully available, without restriction, and from the time of publication. PLOS allows rare exceptions to address legal and ethical concerns. See the PLOS Data Policy and FAQ for detailed information.	Yes - all data are fully available without restriction

A Data Availability Statement describing where the data can be found is required at submission. Your answers to this question constitute the Data Availability Statement and **will be published in the article**, if accepted.<br><br>**Important:** Stating 'data available on request from the author' is not sufficient. If your data are only available upon request, select 'No' for the first question and explain your exceptional situation in the text box.<br><br>Do the authors confirm that all data underlying the findings described in their manuscript are fully available without restriction?	
**Describe where the data may be found in full sentences. If you are copying our sample text, replace any instances of XXX with the appropriate details.**<br><br>• If the data are **held or will be held in a public repository**, include URLs, accession numbers or DOIs. If this information will only be available after acceptance, indicate this by ticking the box below. For example: *All XXX files are available from the XXX database (accession number(s) XXX, XXX.*).<br>• If the data are all contained **within the manuscript and/or Supporting Information files**, enter the following: *All relevant data are within the manuscript and its Supporting Information files.*<br>• If neither of these applies but you are able to provide **details of access elsewhere**, with or without limitations, please do so. For example:<br><br>*Data cannot be shared publicly because of [XXX]. Data are available from the XXX Institutional Data Access / Ethics Committee (contact via XXX) for researchers who meet the criteria for access to confidential data.*<br><br>*The data underlying the results presented in the study are available from (include the name of the third party*	All relevant data are provided within the manuscript and details of data and its supporting information are available as files.

and contact information or URL).<br>• This text is appropriate if the data are owned by a third party and authors do not have permission to share the data.<br><br>* typeset	
Additional data availability information:	Tick here if your circumstances are not covered by the questions above and you need the journal’s help to make your data available.

Powered by Editorial Manager® and ProduXion Manager® from Aries Systems Corporation

Manuscript

Click here to download Manuscript Esam et al manuscript 6-20-2020.docx

Analysis of Molecular Markers in the Environmental Strains of *Vibrio Parahaemolyticus* Isolated from the Oyster (*Crassostrea virginica*) and Water Samples of Delaware Bay

Esam Almuhaideb [1,2], Lathadevi K. Chintapenta [3,4], Amanda Abbott [3], Salina Parveen [2], Gulnihal Ozbay [3,*].

[1] Department of Human Ecology, Delaware State University, Dover, Delaware, United States of America

[2] Department of Agriculture, Food and Resource Sciences, University of Maryland Eastern Shore, Princess Anne, Maryland, United States of America

[3] Department of Agriculture and Natural Resources, Delaware State University, Dover, Delaware, United States of America

[4] Department of Biology, University of Wisconsin River Falls, Wisconsin, United States of America

* Corresponding author

E-mail: gozbay@desu.edu (GO)

Abstract

This study ~~is aimed to~~ ~~identify~~ *Vibrio parahaemolyticus* in oyster and seawater samples collected from Delaware Bay from June through October of 2016. CHROMagar™ Vibrio medium was used to identify *V. parahaemolyticus*, in which presumptive *V. parahaemolyticus* colonies display mauve color. The highest mean levels of *V. parahaemolyticus* were 9.63×10^{3} CFU/g and 1.85×10^{3} CFU/mL in the oyster and seawater samples during the month of July. The lowest detectable limit of *V. parahaemolyticus* in oyster and seawater samples was 1.7×10 CFU/g (or mL) during the months of September and October. ~~We have confirmed that~~ *V. parahaemolyticus* levels were positively correlated with temperature in oyster and seawater samples. *V. parahaemolyticus* isolates (n=165) were further characterized by screening the species-specific thermolabile hemolysin (*tlh*) gene, the pathogenic thermostable direct hemolysin (*tdh*)/ thermostable related hemolysin (*trh*) genes, the regulatory transmembrane DNA-binding gene (*toxR*), and *V. parahaemolyticus* metalloprotease (*vpm*) gene. Of the 165 isolates, 137 (83%), 110 (66.7%), and 108 (65%) were *tlh*$^{+}$, *vpm*$^{+}$, and *toxR*$^{+}$, respectively. Among the *V. parahaemolyticus* (*tlh*$^{+}$) isolates, 7 (5.1%) and 15 (10.9%) were *tdh*$^{+}$ and *trh*$^{+}$, respectively, and 24 (17.5%), only oyster isolates, were positive for both genes. Potential pathogenic strains that possessed *tdh* and/or *trh* were notably higher in oyster (39%) than seawater (15.6%) isolates. Occurrence of total *V. parahaemolyticus* (*tlh*$^{+}$) ~~were~~ not necessarily proportional to the potential pathogenic *V. parahaemolyticus*. Co-occurrence of the five genetic markers were observed only among oyster isolates. Coexistence analysis of the gene markers revealed a relatedness potential of *tdh* occurrence with *vpm*. We believe exploring the role of *V. parahaemolyticus* metalloprotease and whether it involved in the toxic activity of the thermostable direct hemolysin (TDH) protein can

be of significance. We expect our project outcomes will provide some foundation for future studies regarding pathogenic *Vibrio* dynamics in relation to environmental quality.

Introduction

Vibrio parahaemolyticus is a Gram-negative, halophilic, pathogenic bacterium that negatively impact aquatic ecosystems and human health [1-3]. They are curved rods, motile with a single polar flagellum and belong to the family *Vibrionaceae*. It is an endemic pathogen in the marine environment that was first identified as a cause of food-borne illness in Japan in 1950. It results in one of the key causes of gastroenteritis leading to diarrhea, headache, vomiting, and abdominal cramps following the consumption of contaminated food or water. In addition, this bacterium can cause septicemia and wound infections [3, 4].

In aquatic ecosystems, organisms like oysters which are filter-feeding mollusks, tend to accumulate different microorganisms from seawater during their filtration [5-7]. Therefore, they are able to accumulate *V. parahaemolyticus* ~~resulting in a concentration~~100-fold higher than the water ~~of the surrounding area~~ [6, 7]. During the warmer months, *V. parahaemolyticus* contamination in oysters ~~might~~ reach 100% [6].

V. parahaemolyticus infections are associated with the consumption of seafood, particularly raw or undercooked oysters, and accounted for 59.5% of laboratory-confirmed Vibrionac[illegible] [8]. In 2006, a total of 177 *V. parahaemolyticus* infections were reported from New York, Oregon, and Washington states, and the laboratory-confirmed cases were over three-folds higher than the average number in all US states during the same period of 2002-2004 [9]. An outbreak of *V. parahaemolyticus* involving three people was reported in Maryland, August 2012 [10]. A multistate outbreak of 16 gastrointestinal illnesses linked to oysters were reported in

2019, and four of them were associated with *V. parahaemolyticus* [11]. Estimated annual cost mean of foodborne illnesses associated with *V. parahaemolyticus* was over US $40 million [12].

V. parahaemolyticus strains possess a *tlh* species specific gene, which codes for the thermolabile hemolysin (TLH) [13, 14]. The virulence of most clinical *V. parahaemolyticus* isolates are associated with the expression of *tdh* (thermostable direct hemolysin (TDH)) and/or *trh* (TDH-related hemolysin-(TRH)) genes [15-17]. Both *tdh*/*trh* genes are associated with β hemolysis on Wagatsuma blood agar, which is known as the Kanagawa Phenomenon, and both have been used as accepted genetic markers for the detection of pathogenic *V. parahaemolyticus* in seafood [18-20]. Although, TDH/TRH proteins are the main pathogenic factors in *V. parahaemolyticus* [21], research also shows that many of the clinical isolates possess neither *tdh* nor *trh* genes indicating the potential presence of other virulence-related factors [22, 23].

Miyoshi et al. and Kim et al. [24, 25] have reported that *V. parahaemolyticus* harbors a *V. parahaemolyticus* metalloprotease (*vpm*) gene that expresses extracellular zinc metalloprotease and shows sufficient proteolytic activity towards type I collagen. A study conducted by Luan et al. [2] indicated that *V. parahaemolyticus* metalloprotease can degrade host tissue and may promote pathogen invasion. On the other hand, metalloprotease has been investigated and found to be significant as a virulence factor among *Vibrio* spp. [25]. Metalloprotease was reported to have an important role on the extracellular cleavage and activation process of the *V. cholerae* enterotoxic hemolysin into mature hemolysin [26-29]. Therefore, exploring the prevalence and co-occurrence of *vpm* and *tdh*/*trh* genes in the environmental strains of *V. parahaemolyticus* can be of significance.

The transmembrane DNA-binding protein, ToxR, is a regulatory protein in *V. parahaemolyticus* that is encoded by *toxR* gene. The ToxR protein is strongly associated with the

upregulation of the gene encoding the virulence toxin TDH [30]. Genome sequencing of pathogenic *V. parahaemolyticus* revealed another virulence factor called type III secretion systems (T3SS), T3SS1and T3SS2, by which bacterial proteins (effectors) are injected directly into host cells [31]. According to Ritchie et al. [32], an infant rabbit model infected with *V. parahaemolyticus* revealed that T3SS2 is essential for intestinal colonization. In addition, T3SS2 is also considered as a prime virulence factor of *V. parahaemolyticus* enterotoxicity [32-34]. Hubbard et al. [35] identified an uncharacterized component of T3SS2 to be critically regulated by ToxR, contrary to what has been reported by Gotoh et al. [36]. Provenzano et al. and Whitaker et al. [37, 38] have reported that the *toxR* gene is very important to the bile resistance in the intestine, and the *toxR* mutant strains have significantly lower minimal bactericidal concentration compared to the wild strains. The study by Whitaker et al. [37] indicated that the *toxR* gene is required for stress tolerance and colonization of *V. parahaemolyticus*. On the other hand, similar to the *tlh* gene, *toxR* can be a reliable gene for the detection of *V. parahaemolyticus*, and many studies have used it as *V. parahaemolyticus* species-specific gene marker [39-41]. Furthermore, studies also indicate that *tlh* and *toxR* genes have a compatible and robust result in terms of reliability and specificity for molecular identification of *V. parahaemolyticus* [13, 42]. Findings and reports from previous literature highlight the important relationships among the *tlh*, *trh*, *tdh*, *vpm* and *toxR* genes in terms of pathogenicity and identification of *V. parahaemolyticus*. Therefore, our study aims to screen the above-mentioned genetic markers to further illustrate the prevalence and patterns of these genetic markers in environmental strains of *V. parahaemolyticus*.

Delaware Bay is the prime oyster ground on the Atlantic coast providing ecological and commercial resources [43]. *V. parahaemolyticus* outbreaks are one of the leading causes for the

closures of commercial shellfish industries on the U.S. east coast [44, 45]. This study was conducted to detect and determine total and potential pathogenic *V. parahaemolyticus* levels in oyster and seawater samples from the Delaware Bay. This study also examined the correlation of *V. parahaemolyticus* levels in oyster and seawater samples in relation to the physico-chemical parameters. Along with the above-mentioned aims, we reported the co-occurrences of the five genetic markers (*tlh*, *tdh*, *trh*, *toxR*, and *vpm*) in the environmental strains of *V. parahaemolyticus*. To the best of our knowledge there are no published studies on the prevalence and co-occurrence of these genetic markers among *V. parahaemolyticus* in the Mid-Atlantic region. Furthermore, the regional variation in ecology of *V. parahaemolyticus* indicates the need of site-specific data, and this study provides a new set of data specific for the Delaware Bay region.

Materials and methods

Study location and sampling

Oysters and seawater samples were collected once a month from June to October 2016 from Bowers Beach (BB) and Lewes, Broadkill (LW) in the Delaware Bay. A third site, Slaughter Beach (SL) was added to this study from August to October 2016 (Fig 1) to assess a wider range of the Delaware Bay area. Ten to twelve oysters from each site were harvested (one site per week) into Ziploc bags (SC Johnson & Sons, Racine, WI, USA), and sub-divided into three groups for biological triplicates (A, B, and C), and placed in an insulated cooler with ice packs to maintain the temperature between 2-10°C [46]. One liter of seawater was collected from each site at the same time. Water quality parameters such as water temperature, salinity, turbidity, dissolved oxygen, pH, and chlorophyll *a* were recorded onsite using YSI 556 Handheld

Multiparameter Instrument (YSI Incorporated, Yellow Springs, OH, USA) to assess the relationship between these parameters and the Colony Forming Units (CFUs) of *Vibrio parahaemolyticus*.

Fig 1. Study locations in Delaware Bay.

Processing of oyster and seawater samples

Ten to twelve oysters were collected from each site and divided into three groups to be analyzed in triplicates. For each replicate 3-4 oysters were cleaned upon arrival at the Aquatic Laboratory using a scrub brush and tap water before they were shucked with sterile knives. Oysters tissues and liquors from each replicate were placed into a sterilized blender jar (Waring Commercial, 7010S), blended for 90 sec at high speed. Twenty-five grams of the blended tissue was diluted with 225 mL of 0.1% Peptone Water (PW) and blended again for 60 sec at high speed to prepare the homogenate. This homogenate was labelled as the first (10^{-1}) dilution. The oyster homogenate and seawater samples from each site were aseptically serial diluted in 0.1% PW to a final dilution of (10^{-6}). One hundred microliters of each dilution [10^{-1} – 10^{-6}] of both seawater and oyster homogenate samples from each site were aseptically spread plated on CHROMagar plates (CHROMagar™ Vibrio, VB912). This assay was performed in technical duplicates and the plates were incubated for 24h at 37°C.

Identification and isolation of *V. parahaemolyticus*

V. parahaemolyticus were identified as ~~the~~ colonies ~~that displayed mauve color~~ on the CHROMagar plates. Each plate with a countable range of 20 to 200 colonies was used to

calculate the Colony Forming Units (CFU). Using the sterile loop, at least 20% of the mauve colonies from each plate were chosen and inoculated into a 1.5 mL micro centrifuge tube of Tryptone Soy Broth (TSB), using aseptic techniques. Bacterial isolates were incubated with shaking (175 rpm) for overnight at 37°C (New Brunswick Scientific I 24 Incubator Shaker Series). Bacterial isolates in the microcentrifuge tubes were then centrifuged at 15,000 rpm for 2 min (Eppendorf Centrifuge 5424), and the supernatant was discarded. Equal amounts (600 µL) of Alkaline Peptone Water (APW) and TSB + [24% Glycerol] were added and the pellet was resuspended and then frozen at -20°C for further molecular analysis. Samples were prepared for PCR by boiling for ten minutes, and immediately chilled (2 min) for cell lysis and DNA release.

Molecular analysis (PCR procedures and conditions)

Presumptive *V. parahaemolyticus* isolates were further typed for the genetic markers *tlh*, *tdh*, *trh*, *toxR*, and *vpm* using five sets of primers previously assessed by Luan et al. and Nordstrom et al. [13, 47]. The PCR reaction mixture (10uL) consisted of 1 µL of the tested DNA template, 2 µL of the 5x loading buffer (Taq Buffer; PROMEGA, USA), 0.1 µL of Taq polymerase (Taq; PROMEGA; USA), 0.4 µL of 2.5 mM deoxynucleotide mix, 0.2 µL of each forward and reverse primers (IDT; USA), and 6.3 µL of nuclease free water. The amplification conditions of *tlh*, *tdh*, *trh*, *toxR*, and *vpm* genes are shown in (Table 1), and PCR reactions were performed using S1000; Thermal Cycler; Bio-Rad. Negative and positive controls were used in this study as follows: (a) 1 µL of nuclease free water as no DNA template (negative control); (b) 1 µL of *V. parahaemolyticus* as a DNA template (positive control).

Table 1. PCR conditions and primers sequences used in this study.

	Genes		Primers Sequences
	tlh/tdh/trh	***toxR/vpm***	
			F-*tlh*: ACTCAACACAAGAAGAGATCGACAA
**Cycles**	30	35	R-*tlh*: GATGAGCGGTTGATGTCCAA
			F-*tdh*: TCCCTTTTCCTGCCCCC
**Denaturation temp**	95°C/30sec	94°C/1 min	R-*tdh*: CGCTGCCATTGTATAGTCTTTATC
			F-*trh*: TTGCTTTCAGTTTGCTATTGGCT
			R-*trh*: TGTTTACCGTCATATAGGCGCTT
**Annealing temp**	60°C/45sec	58°C/1 min	*toxR*-4: GTCTTCTGACGCAATCGTTG
			toxR-7: ATACGAGTGGTTGCTGTCATG
**Extension temp**	68°C/1 min	72°C/1 min	*vpm*1: CAGCTACCGAAACAGACGCTA
			*vpm*2: TCCTATCGAGGACTCTCTCAAC

Data analysis

For statistical analysis, the Log$_{10}$ transformation of the CFU values of presumptive *V. parahaemolyticus* was performed to normalize the data, and the significance level (*P*-value) of 0.05 was used. Spearman's rank correlation analysis was performed to measure the relationship between *V. parahaemolyticus* levels [Log$_{10}$ CFU/g (or mL)] and the parameters affecting water quality (temperature, salinity, dissolved oxygen (DO), pH, turbidity, and chlorophyll *a*). Independent samples t-test was used to determine whether *V. parahaemolyticus* levels [Log$_{10}$ CFU/g (or mL)] among the sample types (oyster and seawater) were significantly different. Statistical analysis was performed using IBM SPSS Statistic software (version 26).

Results and discussion

Physico-chemical water quality parameters.

Physico-chemical water quality parameters (Table 2) show that water temperatures ranged from 14.63 °C (LW, October) to 28 °C (BB, August). Salinity levels were in the range of 5.37ppt (LW, October) to 32ppt (SL, August). The lowest and highest ranges for dissolved oxygen (DO) (3.12 to 8.23 mg/L) were recorded during the months of August and October from BB and LW sites, respectively. The minimum pH value of 6.44 (LW) and maximum of 8.82 (BB) was observed during the month of October. In terms of turbidity and chlorophyll *a*, the minimum and maximum levels ranged from 19 to 55.35 NTU/FTU and 0.134 to 1.174 µg/L, respectively. Notably, at the LW site and during the month of October, water quality parameters displayed the lowest range of water temperature (14.63°C), minimum level of salinity (5.37ppt), highest range of dissolved oxygen (8.23mg/L), and minimum pH value of (6.44). The seasonal variation between temperature and dissolved oxygen reported by Davis et al. [48] shows that the median of temperature (°C) is inversely correlated with the median of dissolve oxygen (mg/L). Parveen et al. [49] have also reported the lowest dissolved oxygen level (5.3 mg/L), and the highest temperature (29.4°C) during the month of August. This shows that temperature is inversely correlated with dissolved oxygen concentrations [50].

Table 2. Physico-chemical water quality parameters in relation to study sites and date of collection.

Site	Date	Temp °C	Salinity ppt	Turbidity NTU/FTU	Dissolved Oxygen mg/L	Chlorophyll *a* µg/L	pH
	06/21/2016	24.18	20.0	29.0	6.3	1.2	8.18
	07/19/2016	27.74	27.0	19.0	3.9	0.7	7.88
**BB**	08/02/2016	28.00	25.0	43.5	3.1	0.8	7.88
	09/13/2016	23.67	26.0	45.1	4.0	1.1	8.04
	10/17/2016	17.91	25.8	55.1	8.1	0.2	8.82
	06/07/2016	22.7	23.0	29.0	3.7	0.3	7.2
	07/06/2016	22.98	32.0	33.0	4.3	0.4	7.84
**LW**	08/08/2016	26.43	25.0	40.8	3.4	0.2	7.55
	09/06/2016	21.32	24.0	39.0	3.4	0.1	7.75
	10/10/2016	14.63	5.40	54.8	8.2	0.5	6.44
	08/30/2016	26.74	32.0	55.4	4.0	0.8	8.06
**SL**	09/26/2016	20.82	26.5	55.1	4.7	0.3	7.31
	10/24/2016	14.68	16.6	20.0	7.8	0.3	7.44

BB: Bowers Beach; LW: Lewes, Broadkill; SL: Slaughter Beach

Concentration of *V. parahaemolyticus* in oyster and seawater samples

The highest mean levels of presumptive *V. parahaemolyticus* ~~was~~ 9.63×10^3 CFU/g in the oyster samples during the month of July from BB site. This was higher than *V. parahaemolyticus*

(CFU) levels (6.0×10^2 CFU/g) detected by direct plating-colony hybridization procedure in Maryland Chesapeake Bay oysters [49]. According to the U.S. Food and Drug Administration (FDA) regulations and guidance, *V. parahaemolyticus* levels (Kanagawa positive or negative) in this study did not exceed the safety limits ($\geq 1\times10^4$ CFU/g) [51]. Clearly, all presumptive *V. parahaemolyticus* (CFU) levels, agree well with the strong correlations between water temperature and *V. parahaemolyticus* densities that were reported in the literature [49, 52-54], indicating that *V. parahaemolyticus* levels increases with the rise of temperature and vice versa (Table 3). CFU counts of *V. parahaemolyticus* from seawater samples were notably lower than oyster samples (Table 3), demonstrating that oysters can concentrate the *Vibrio* species higher than 10-fold compared to the water of the surrounding areas [6, 7]. However, Independent samples t-test indicated that there was no statistically significant difference in mean *V. parahaemolyticus* Log_{10} CFU/g (or ml) values between sample types (oyster – seawater), $t(24) = 1.159$, $P = 0.258$ ($P > 0.05$). Seawater samples from LW in July, with the highest range of salinity, demonstrated low CFU/mL counts compared to BB during the same month indicating that there are parameters other than temperature that may have affected the growth *V. parahaemolyticus* (Urquhart et al., 2016). During the month of October, *V. parahaemolyticus* levels were undetectable (<10 CFU/g (or mL)) in both oyster and seawater samples from LW and SL sites. However, the oyster and seawater samples at site BB showed 1.7×10 and 3.3×10 CFU/g or mL respectively. Overall, the lowest detectable limit of *V. parahaemolyticus* in oyster and seawater samples was 1.7×10 CFU/g (or mL). Figs 2 and 3 demonstrate the Log10 CFU/g (or mL) levels of *V. parahaemolyticus* in relation to collection time, study site, and water parameters (Temperature and Salinity) for oyster and seawater samples, respectively.

Table 3. Averages of *V. parahaemolyticus* CFU/g (or mL) in relation to sample type, study site, and collection time.

	BB-OY	BB-W	LW-OY	LW-W	SL-OY	SL-W
**June**	2017	33	367	83	-[b]	-[b]
**July**	9633	1100	1850	167	-[b]	-[b]
**Aug**	980	617	1133	117	117	20
**Sep**	417	<10[a]	<10[a]	17	17	33
**Oct**	17	33	<10[a]	<10[a]	<10[a]	<10[a]

OY: Oyster; W: Water; BB: Bowers Beach; LW: Lewes, Broadkill; SL: Slaughter Beach.

[a] (Not detectable); [b] (No sample collection)

Fig 2. Average *Vibrio parahaemolyticus* levels (Log$_{10}$ CFU/g) in oyster samples in relation to collection time, study sites, and water parameters (Temperature and Salinity). Bowers Beach (BB) - Lewes, Broadkill (LW) - Slaughter Beach (SL).

Fig 3. Average *Vibrio parahaemolyticus* levels (Log10 CFU/mL) in seawater samples in relation to time collection, study sites, and water parameter (Temperature and Salinity). Bowers Beach (BB) - Lewes, Broadkill (LW) - Slaughter Beach (SL).

Spearman's rank correlation analysis shows that temperature is positively and significantly ($P < 0.05$) correlated to the total *V. parahaemolyticus* (Log$_{10}$ CFU/g or mL) levels in oyster and seawater samples (Table 4), which is in agreement with the previous studies [49, 52-54]. Salinity has no significant correlation with total *V. parahaemolyticus* (Log$_{10}$ CFU/g or mL) levels in oyster and seawater samples (Table 4). Several studies have reported conflicting

results regarding the correlations between the abundance of *V. parahaemolyticus* and salinity. Studies conducted by Tey et al., Yu et al., Martinez-Urtaza et al., and Zimmerman et al. [55-58] have found a correlation between salinity and abundance of *V. parahaemolyticus*, while Parveen et al., Froelich et al., Cruz et al., Johnson et al., Kirs et al., and Deepanjali et al. [49, 59-63] have not. Thus, the insignificance of salinity on the abundance of *V. parahaemolyticus* identified in this study cannot be generalized. No significant correlation was found between dissolved oxygen/turbidity and the abundance of total *V. parahaemolyticus* (Table 4). This result is in contrast to what have been reported from studies conducted in the Chesapeake Bay [48, 49]. Both pH and Chlorophyll *a* did not significantly correlate with total *V. parahaemolyticus* (Log_{10} CFU/g or mL) levels (Table 4), and this is consistent with the previous studies from Mid-Atlantic region [48, 49, 61].

Table 4. Correlation between *V. parahaemolyticus* Log_{10} CFU level and water parameters.

Spearman's correlation coefficient, *r*	Oyster (Sig.)	Seawater (Sig.)
Temp	**.777 [a] (.002)**	**.639 [a] (.019)**
Salinity	.416 (.157)	.423 (.149)
Turbidity	-.393 (.184)	-.306 (.309)
DO mg/l	-.368 (.216)	-.528 (.064)
pH	.416 (.158)	.142 (.644)
Chlorophyll *a*	.509 (.076)	.033 (.914)

[a] Correlation is significant at the 0.05 level

Sig. (2-tailed)

Molecular identification and characterization of *V. parahaemolyticus*

A total of 165 presumptive *V. parahaemolyticus* isolates (mauve colored on CHROM agar) were further examined for the presence of the species-specific gene (*tlh*), and 137 (83%) were confirmed to be *V. parahaemolyticus* (Table 5). Previous investigation by Luan et al. [13] reveled that primers targeting the *tlh*, *toxR*, and *vpm* genes were (100%) specific for *V. parahaemolyticus* strains. The lower occurrence of *vpm* (66.7%) and *toxR* (65.5%) genes compared to *tlh* (83%) gene in this study (Table 6 and Fig 4), suggests that *tlh* gene may occasionally produce false positive results, as a gene similar to *tlh* may occur in other *Vibrio* species specifically *V. alginolyticus* [5, 64]. Yet, regulatory authorities use *tlh* gene as a marker to assess the counts of *V. parahaemolyticus* and reinforce actions to control the outbreaks [65]. This study also showed that (11.7%) of the confirmed *V. parahaemolyticus* possessed only *tlh* gene (Fig 5). In contrast, *toxR* and/or *vpm* genes were only present in coexistence with *tlh*, *tdh*, and/or *trh* (Fig 5), suggest that *toxR* and *vpm* may be more sensitive in detecting *V. parahaemolyticus*. High percentage of *V. parahaemolyticus* (*tlh*$^+$) were observed among oysters (90.5%) compered to seawater (65.3%) samples (Table 5). At LW and SL sites, *V. parahaemolyticus* were not-detectable (ND) during the month of October (Table 5). About half of the confirmed *V. parahaemolyticus* colonies isolated from seawater (43.8%) possessed only *tlh* gene indicating the necessity of other gene markers such as *toxR* and *vpm* for *V. parahaemolyticus* strains to survive the internal conditions and colonize oyster gut [2, 37].

Table 5. Occurrence of presumptive and confirmed (*tlh*+) *V. parahaemolyticus* isolates on CHROMagar.

Site	Month	Total Presumptive *Vp.* (OY, W)	Total Confirmed *Vp.* ***tlh***$^{+}$	Confirmed *Vp.* Oyster ***tlh***$^{+}$	Confirmed *Vp.* Water ***tlh***$^{+}$
**BB**	June	16 (14, 2)	15 (93.8%)	13 (92.9%)	2 (100%)
	July	36 (20, 16)	34 (94.4%)	20 (100%)	14 (87.5%)
	August	22 (15, 7)	18 (81.8%)	14 (93.3%)	4 (57.1%)
	September	7 (7, 0)	7 (100%)	7 (100%)	0
	October	3 (2, 1)	1 (33.3%)	1 (50%)	0
**LW**	June	10 (10, 0)	8 (80%)	8 (80%)	0
	July	40 (33, 7)	36 (90%)	31 (93.9%)	5 (71.4%)
	August	16 (11, 5)	11 (68.8%)	7 (63.6%)	4 (80%)
	September	1 (0, 1)	0	0	0
	October	ND	ND	ND	ND
**SL**	August	12 (3, 9)	6 (50%)	3 (100%)	3 (33.3%)
	September	2 (1, 1)	1 (50%)	1 (100%)	0
	October	ND	ND	ND	ND
	**Total**	165 (116, 49)	137 (83%)	105 (90.5%)	32 (65.3%)

ND: Not detectable; OY: Oyster; W: Water; BB: Bowers Beach; LW: Lewes, Broadkill; SL: Slaughter Beach.

Table 6. Prevalence of *V. parahaemolyticus* genetic markers.

Site	Month	*tlh*	*tdh*	*trh*	*tdh/trh*	*toxR*	*vpm*
		OY, W	OY, W	OY, W	OY, W	OY, W	OY, W
**BB**	June	13 , 2	1 , 0	5 , 1	2 , 0	12 , 0	11 , 0
	July	20 , 14	2 , 2	3 , 1	1 , 0	14 , 4	20 , 7
	August	14 , 4	0 , 0	1 , 0	0 , 0	14 , 1	12 , 0
	September	7 , ND	0 , ND	0 , ND	0 , ND	6 , ND	6 , ND
	October	1 , 0	0 , 0	0 , 0	0 , 0	1 , 0	0 , 0
**LW**	June	8 , 0	1 , 0	0 , 0	0 , 0	8 , 0	7 , 0
	July	31 , 5	1 , 0	3 , 1	21 , 0	29 , 3	31 , 3
	August	7 , 4	0 , 0	0 , 0	0 , 0	7 , 2	7 , 2
	September	ND , 0	ND , 0	ND , 0	ND , 0	ND , 0	ND , 0
	October	ND , ND	ND , ND	ND , ND	ND , ND	ND , ND	ND , ND
**SL**	August	3 , 3	0 , 0	0 , 0	0 , 0	3 , 3	2 , 2
	September	1 , 0	0 , 0	0 , 0	0 , 0	1 , 0	0 , 0
	October	ND , ND	ND , ND	ND , ND	ND , ND	ND , ND	ND , ND
Total		105, 32	5 , 2	12 , 3	24 , 0	95 , 13	96 , 14

ND: Not detectable; OY: Oyster; W: Water; BB: Bowers Beach; LW: Lewes, Broadkill; SL: Slaughter Beach.

Fig 4. Occurrence and genes frequency among *V. parahaemolyticus* isolates (n=165) from oyster and seawater samples.

Fig 5. Genes coexistence among total confirmed *V. parahaemolyticus* isolates.

Among *V. parahaemolyticus* (*tlh*$^+$), 22.6% and 28.5% were positive for *tdh* and *trh* respectively (Table 6). The low occurrence of *tdh* and *trh* agrees with the low level of these genes among environmental and food isolates reported in the literature [49, 66-68]. Isolates that possessed *tdh, trh,* or both *tdh*/*trh* genes account for 33.5% of *V. parahaemolyticus* (*tlh*$^+$) and were notably higher in oyster (39%) than seawater (15.6%) (Table 6). More than half (61%) of *V. parahaemolyticus* (*tdh*$^+$/*trh*$^+$) isolated from oysters were detected at LW site on July (Table

6). Occurrence of *tdh* and/or *trh* positive *V. parahaemolyticus* was not observed among the study sites during September and October (Table 6). This observation highlights the importance of understanding the dynamics and seasonal variations of pathogenic *V. parahaemolyticus* in Delaware Bay. The high frequency of *trh* gene (n = 39) compared to the *tdh* gene (n = 31) agrees well with its occurrence in Gulf Coast and Chesapeake Bay oysters [49], and in South Carolina [69]. The co-occurrence of both *tdh*/*trh* genes was observed in 17.5% and 52% of total and potential pathogenic *V. parahaemolyticus*, respectively, and they were only among oyster isolates (Table 6). This is in contrast with the results reported by Williams et al. [70], in which *V. parahaemolyticus* ~~possess~~ both *tdh*/*trh* genes were observed more frequently among the water isolates. *V. parahaemolyticus* (*tlh*$^+$) as illustrated in Table 6 were not necessarily proportional to the potential pathogenic *V. parahaemolyticus*, and this agrees well with the previous studies ~~by Klein and Lovell and Williams et al~~. [69, 70].

Occurrence ~~frequency~~ of the five genetic markers showed similar patterns among oyster and seawater isolates (Fig 4). The co-occurrence of the five genetic markers among oyster isolates tested were dominated (43.9%) by *tlh*, *toxR* and *vpm* pattern followed by the coexistence of all five genetic markers (18.9%) (Fig 6). On the other hand, co-occurrence of *tlh*, *toxR*, and *vpm* were the second prevalent pattern among seawater isolates (Fig 7). Interestingly and similar to the simultaneous occurrences of *tdh*/*trh*, the coexistence of the five genetic markers were observed only among oyster isolates (Figs 6 and 7). Variation of gene patterns among the examined isolates suggest the variation of *V. parahaemolyticus* clones that inhabit Delaware Bay. This study also revealed the relatedness potential of *tdh* occurrence with *vpm*, as Figs 5, 6, and 7 demonstrated that whenever there is a (*tdh*) there is a (*vpm*) but not vice versa. This indicates the importance of understanding the role of *V. parahaemolyticus*

metalloprotease and whether it involved in the toxic activity of the thermostable direct hemolysin (TDH) protein as is the case with *V. cholerae* enterotoxic hemolysin [26-29].

Fig 6. Genes coexistence among confirmed *V. parahaemolyticus* isolated from oyster samples.

Fig 7. Genes coexistence among confirmed *V. parahaemolyticus* isolated from seawater samples.

Conclusion

This study assessed *V. parahaemolyticus* CFU/g (or mL) levels to evaluate the bacteriological conditions of seawater and oyster samples collected from Delaware Bay. *V. parahaemolyticus* levels in oyster samples were notably higher than seawater samples. Occurrence of the virulent genes (*tdh* and/or *trh*) in oyster samples were notably higher. The low occurrence of *V. parahaemolyticus* isolates that possessed *tdh*, *trh*, *toxR*, and/or *vpm* genes in seawater samples compared to oysters confirm the significance of the bioaccumulation processed by oysters as a natural nursery for potential pathogenic *V. parahaemolyticus*. Utilizing *V. parahaemolyticus* metalloprotease gene (*vpm*) as species-specific gene may provide more accurate results when assessing the prevalence and abundance of pathogenic *V. parahaemolyticus*, and a better understanding of the proportional correlation between the total and potentially pathogenic *V. parahaemolyticus*. The variation among *V. parahaemolyticus* isolates that we have reported indicates the difference in growth rates among Delaware Bay oysters.

Future studies may focus on conducting whole genome sequencing for the *V. parahaemolyticus* isolates to identify the coexistence of the virulent and virulence related genes reported in the literature and illustrate the genetic diversity among *V. parahaemolyticus* isolates inhibiting Delaware Bay. Future studies may also focus on the role of *V. parahaemolyticus* metalloprotease on the toxic activity of TDH. This study provided informative data on oyster-*Vibrio* contamination factors that can be applied to the risk management programs. We expect our project outcomes will provide some foundation for future studies regarding pathogenic *Vibrio* dynamics in relation to environmental quality.

Acknowledgement

We would like to thank Dr. Bettina C. Taylor for valuable comments and editing. We also thank Dr. Gary Richards of USDA ARS for his valuable discussion on our findings. This study was funded by USDA-NIFA (2014-38821-22430); NSF-EPSCoR Program (EPS-1301765); CIBER at Delaware State University.

References

1. Lee CT, Chen IT, Yang YT, Ko TP, Huang YT, Huang JY, et al. The opportunistic marine pathogen Vibrio parahaemolyticus becomes virulent by acquiring a plasmid that expresses a deadly toxin. Proc Natl Acad Sci U S A. 2015;112(34):10798-803.

2. Luan X, Chen J, Zhang XH, Li Y, Hu G. Expression and characterization of a metalloprotease from a Vibrio parahaemolyticus isolate. Can J Microbiol. 2007;53(10):1168-73.

3. Morris JG, Jr., Black RE. Cholera and other vibrioses in the United States. N Engl J Med. 1985;312(6):343-50.

4. Iwamoto M, Ayers T, Mahon BE, Swerdlow DL. Epidemiology of seafood-associated infections in the United States. Clin Microbiol Rev. 2010;23(2):399-411.

5. Klein SL, Gutierrez West CK, Mejia DM, Lovell CR. Genes similar to the Vibrio parahaemolyticus virulence-related genes tdh, tlh, and vscC2 occur in other vibrionaceae species isolated from a pristine estuary. Appl Environ Microbiol. 2014;80(2):595-602.

6. Morris JG, Jr. Cholera and other types of vibriosis: a story of human pandemics and oysters on the half shell. Clin Infect Dis. 2003;37(2):272-80.

7. DePaola A, Hopkins LH, Peeler JT, Wentz B, McPhearson RM. Incidence of Vibrio parahaemolyticus in U.S. coastal waters and oysters. Appl Environ Microbiol. 1990;56(8):2299-302.

8. Centers for Disease Control and Prevention FDASNF. FoodNet 2015 Surveillance Report (Final Data). Atlanta, GA: Centers for Disease Control and Prevention, Foodborne Diseases Active Surveillance Network (FoodNet), Services USDoHaH; 2017.

9. Centers for Disease Control and Prevention. Vibrio parahaemolyticus infections associated with consumption of raw shellfish--three states, 2006. MMWR Morbidity and mortality weekly report. 2006;55(31):854.

10. Haendiges J, Rock M, Myers RA, Brown EW, Evans P, Gonzalez-Escalona N. Pandemic Vibrio parahaemolyticus, Maryland, USA, 2012. Emerg Infect Dis. 2014;20(4):718-20.

11. Centers for Disease Control and Prevention NCEZID. Multistate Outbreak of Gastrointestinal Illnesses Linked to Oysters Imported from Mexico. Atlanta, GA: Centers for Disease Control and Prevention, National Center for Emerging and Zoonotic Infectious Diseases, Division of Foodborne W, and Environmental Diseases; 2019 6/21/2019.

12. U.S. Department of Agriculture ERS. Cost estimates of foodborne illnesses: Cost of foodborne illness estimates for Vibrio parahaemolyticus. Washington, DC2014.

13. Luan XY, Chen JX, Zhang XH, Jia JT, Sun FR, Li Y. Comparison of different primers for rapid detection of Vibrio parahaemolyticus using the polymerase chain reaction. Lett Appl Microbiol. 2007;44(3):242-7.

14. Bej AK, Patterson DP, Brasher CW, Vickery MC, Jones DD, Kaysner CA. Detection of total and hemolysin-producing Vibrio parahaemolyticus in shellfish using multiplex PCR amplification of tl, tdh and trh. J Microbiol Methods. 1999;36(3):215-25.

15. Okuda J, Ishibashi M, Abbott SL, Janda JM, Nishibuchi M. Analysis of the thermostable direct hemolysin (tdh) gene and the tdh-related hemolysin (trh) genes in urease-positive strains of Vibrio parahaemolyticus isolated on the West Coast of the United States. J Clin Microbiol. 1997;35(8):1965-71.

16. Shirai H, Ito H, Hirayama T, Nakamoto Y, Nakabayashi N, Kumagai K, et al. Molecular epidemiologic evidence for association of thermostable direct hemolysin (TDH) and TDH-related hemolysin of Vibrio parahaemolyticus with gastroenteritis. Infect Immun. 1990;58(11):3568-73.

17. Taniguchi H, Ohta H, Ogawa M, Mizuguchi Y. Cloning and expression in Escherichia coli of Vibrio parahaemolyticus thermostable direct hemolysin and thermolabile hemolysin genes. J Bacteriol. 1985;162(2):510-5.

18. Wagley S, Koofhethile K, Rangdale R. Prevalence and potential pathogenicity of Vibrio parahaemolyticus in Chinese mitten crabs (Eriocheir sinensis) harvested from the River Thames estuary, England. J Food Prot. 2009;72(1):60-6.

19. Tada J, Ohashi T, Nishimura N, Shirasaki Y, Ozaki H, Fukushima S, et al. Detection of the thermostable direct hemolysin gene (tdh) and the thermostable direct hemolysin-related hemolysin gene (trh) of Vibrio parahaemolyticus by polymerase chain reaction. Mol Cell Probes. 1992;6(6):477-87.

20. Honda T, Chearskul S, Takeda Y, Miwatani T. Immunological methods for detection of Kanagawa phenomenon of Vibrio parahaemolyticus. J Clin Microbiol. 1980;11(6):600-3.

21. Yamazaki W, Kumeda Y, Misawa N, Nakaguchi Y, Nishibuchi M. Development of a loop-mediated isothermal amplification assay for sensitive and rapid detection of the tdh and trh genes of Vibrio parahaemolyticus and related Vibrio species. Appl Environ Microbiol. 2010;76(3):820-8.

22. García K, Torres R, Uribe P, Hernández C, Rioseco ML, Romero J, et al. Dynamics of clinical and environmental Vibrio parahaemolyticus strains during seafood-related summer diarrhea outbreaks in southern Chile. Appl Environ Microbiol. 2009;75(23):7482-7.

23. Honda S, Goto I, Minematsu I, Ikeda N, Asano N, Ishibashi M, et al. Vibrio parahaemolyticus infectious disease caused by Kanagawa phenomenon-negative O3: K6 originated from Maldives. Kansenshogaku zasshi The Journal of the Japanese Association for Infectious Diseases. 1987;61(9):1070-8.

24. Miyoshi S-i, Nitanda Y, Fujii K, Kawahara K, Li T, Maehara Y, et al. Differential gene expression and extracellular secretion of the collagenolytic enzymes by the pathogen Vibrio parahaemolyticus. FEMS Microbiol Lett. 2008;283(2):176-81.

25. Kim S-K, Yang J-Y, Cha J. Cloning and sequence analysis of a novel metalloprotease gene from Vibrio parahaemolyticus 04. Gene. 2002;283(1-2):277-86.

26. Benitez JA, Silva AJ. Vibrio cholerae hemagglutinin (HA)/protease: an extracellular metalloprotease with multiple pathogenic activities. Toxicon. 2016;115:55-62.

27. Binesse J, Delsert C, Saulnier D, Champomier-Verges M-C, Zagorec M, Munier-Lehmann H, et al. Metalloprotease vsm is the major determinant of toxicity for extracellular products of Vibrio splendidus. Appl Environ Microbiol. 2008;74(23):7108-17.

28. Nagamune K, Yamamoto K, Naka A, Matsuyama J, Miwatani T, Honda T. In vitro proteolytic processing and activation of the recombinant precursor of El Tor cytolysin/hemolysin (pro-HlyA) of Vibrio cholerae by soluble hemagglutinin/protease of V. cholerae, trypsin, and other proteases. Infect Immun. 1996;64(11):4655-8.

29. Booth B, Boesman-Finkelstein M, Finkelstein R. Vibrio cholerae hemagglutinin/protease nicks cholera enterotoxin. Infect Immun. 1984;45(3):558-60.

30. Lin Z, Kumagai K, Baba K, Mekalanos J, Nishibuchi M. Vibrio parahaemolyticus has a homolog of the Vibrio cholerae toxRS operon that mediates environmentally induced regulation of the thermostable direct hemolysin gene. J Bacteriol. 1993;175(12):3844-55.

31. Makino K, Oshima K, Kurokawa K, Yokoyama K, Uda T, Tagomori K, et al. Genome sequence of Vibrio parahaemolyticus: a pathogenic mechanism distinct from that of V cholerae. The Lancet. 2003;361(9359):743-9.

32. Ritchie JM, Rui H, Zhou X, Iida T, Kodoma T, Ito S, et al. Inflammation and disintegration of intestinal villi in an experimental model for Vibrio parahaemolyticus-induced diarrhea. PLoS Pathog. 2012;8(3):e1002593.

33. Hiyoshi H, Kodama T, Iida T, Honda T. Contribution of Vibrio parahaemolyticus virulence factors to cytotoxicity, enterotoxicity, and lethality in mice. Infect Immun. 2010;78(4):1772-80.

34. Park K-S, Ono T, Rokuda M, Jang M-H, Okada K, Iida T, et al. Functional characterization of two type III secretion systems of Vibrio parahaemolyticus. Infect Immun. 2004;72(11):6659-65.

35. Hubbard TP, Chao MC, Abel S, Blondel CJ, Zur Wiesch PA, Zhou X, et al. Genetic analysis of Vibrio parahaemolyticus intestinal colonization. Proceedings of the National Academy of Sciences. 2016;113(22):6283-8.

36. Gotoh K, Kodama T, Hiyoshi H, Izutsu K, Park K-S, Dryselius R, et al. Bile acid-induced virulence gene expression of Vibrio parahaemolyticus reveals a novel therapeutic potential for bile acid sequestrants. PloS one. 2010;5(10).

37. Whitaker WB, Parent MA, Boyd A, Richards GP, Boyd EF. The Vibrio parahaemolyticus ToxRS regulator is required for stress tolerance and colonization in a novel orogastric streptomycin-induced adult murine model. Infect Immun. 2012;80(5):1834-45.

38. Provenzano D, Schuhmacher DA, Barker JL, Klose KE. The Virulence Regulatory Protein ToxR Mediates Enhanced Bile Resistance in Vibrio cholerae and Other PathogenicVibrio Species. Infect Immun. 2000;68(3):1491-7.

39. Lo C, Leung P, Yip S, To T, Ng T, Kam K. Rapid detection of pathogenic Vibrio parahaemolyticus by a sensitive and specific duplex PCR- hybridization probes assay using LightCycler. J Appl Microbiol. 2008;105(2):575-84.

40. Croci L, Suffredini E, Cozzi L, Paniconi M, Ciccaglioni G, Colombo MM. Evaluation of different polymerase chain reaction methods for the identification of Vibrio parahaemolyticus strains isolated by cultural methods. J AOAC Int. 2007;90(6):1588-97.

41. Kim YB, Okuda J, Matsumoto C, Takahashi N, Hashimoto S, Nishibuchi M. Identification of Vibrio parahaemolyticus strains at the species level by PCR targeted to the toxR gene. J Clin Microbiol. 1999;37(4):1173-7.

42. Taiwo M, Baker-Austin C, Powell A, Hodgson E, Natǹs OB, Walker DI. Comparison of toxR and tlh based PCR assays for Vibrio parahaemolyticus. Food Control. 2017;77:116-20.

43. Canzonier WJ. Delaware Bay Oyster Culture – Past, Present and Potential Future. In: Agriculture/Seafood, editor.: N J Aquaculture Association; 2005.

44. Marquis ND, Record NR, Robledo JAF. Survey for protozoan parasites in Eastern oysters (Crassostrea virginica) from the Gulf of Maine using PCR-based assays. Parasitol Int. 2015;64(5):299-302.

45. DePaola A, Jones JL, Woods J, Burkhardt W, Calci KR, Krantz JA, et al. Bacterial and viral pathogens in live oysters: 2007 United States market survey. Appl Environ Microbiol. 2010;76(9):2754-68.

46. Interstate Shellfish Sanitation Conference, National Shellfish Sanitation Program: Guide for the Control of Molluscan Shellfish: Shellfish Laboratory Evaluation Checklist for PCR Microbiology, (2017).

47. Nordstrom JL, Vickery MC, Blackstone GM, Murray SL, DePaola A. Development of a multiplex real-time PCR assay with an internal amplification control for the detection of total and pathogenic Vibrio parahaemolyticus bacteria in oysters. Appl Environ Microbiol. 2007;73(18):5840-7.

48. Davis BJ, Jacobs JM, Davis MF, Schwab KJ, DePaola A, Curriero FC. Environmental determinants of Vibrio parahaemolyticus in the Chesapeake Bay. Appl Environ Microbiol. 2017;83(21):e01147-17.

49. Parveen S, Hettiarachchi KA, Bowers JC, Jones JL, Tamplin ML, McKay R, et al. Seasonal distribution of total and pathogenic Vibrio parahaemolyticus in Chesapeake Bay oysters and waters. Int J Food Microbiol. 2008;128(2):354-61.

50. National Ocean Service Education. Estuaries: Dissolved Oxygen: National Oceanic and Atmospheric Administration 2020 [Available from: https://oceanservice.noaa.gov/education/kits/estuaries/media/supp_estuar10d_disolvedox.html.

51. U.S. Food and Drug Administration, Center for Food Safety and Applied Nutrition: Fish and Fishery Products Hazards and Controls Guidance Fourth Edition, Appendix 5: FDA and EPA Safety Levels in Regulations and Guidance, (2020).

52. Urquhart EA, Jones SH, Jong WY, Schuster BM, Marcinkiewicz AL, Whistler CA, et al. Environmental conditions associated with elevated Vibrio parahaemolyticus concentrations in Great Bay Estuary, New Hampshire. PloS one. 2016;11(5).

53. Takemura AF, Chien DM, Polz MF. Associations and dynamics of Vibrionaceae in the environment, from the genus to the population level. Frontiers in microbiology. 2014;5:38.

54. Ozbay G, Karuna Chintapenta L, Lingham T, Lumor S, Lee J-l, Taylor B, et al. Delaware Inland Bays and Market Oyster (Crassostrea virginica) Quality for Consumption. J Food Qual. 2018;2018.

55. Tey YH, Jong K-J, Fen S-Y, Wong H-C. Occurrence of Vibrio parahaemolyticus, Vibrio cholerae, and Vibrio vulnificus in the aquacultural environments of Taiwan. J Food Prot. 2015;78(5):969-76.

56. Yu W-T, Jong K-J, Lin Y-R, Tsai S-e, Tey YH, Wong H-c. Prevalence of Vibrio parahaemolyticus in oyster and clam culturing environments in Taiwan. Int J Food Microbiol. 2013;160(3):185-92.

57. Martinez-Urtaza J, Lozano-Leon A, Varela-Pet J, Trinanes J, Pazos Y, Garcia-Martin O. Environmental determinants of the occurrence and distribution of Vibrio parahaemolyticus in the rias of Galicia, Spain. Appl Environ Microbiol. 2008;74(1):265-74.

58. Zimmerman A, DePaola A, Bowers J, Krantz J, Nordstrom J, Johnson C, et al. Variability of total and pathogenic Vibrio parahaemolyticus densities in northern Gulf of Mexico water and oysters. Appl Environ Microbiol. 2007;73(23):7589-96.

59. Froelich B, Ayrapetyan M, Fowler P, Oliver J, Noble R. Development of a matrix tool for the prediction of Vibrio species in oysters harvested from North Carolina. Appl Environ Microbiol. 2015;81(3):1111-9.

60. Cruz C, Hedderley D, Fletcher G. Long-term study of Vibrio parahaemolyticus prevalence and distribution in New Zealand shellfish. Appl Environ Microbiol. 2015;81(7):2320-7.

61. Johnson CN, Bowers JC, Griffitt KJ, Molina V, Clostio RW, Pei S, et al. Ecology of Vibrio parahaemolyticus and Vibrio vulnificus in the coastal and estuarine waters of Louisiana, Maryland, Mississippi, and Washington (United States). Appl Environ Microbiol. 2012;78(20):7249-57.

62. Kirs M, Depaola A, Fyfe R, Jones J, Krantz J, Van Laanen A, et al. A survey of oysters (Crassostrea gigas) in New Zealand for Vibrio parahaemolyticus and Vibrio vulnificus. Int J Food Microbiol. 2011;147(2):149-53.

63. Deepanjali A, Kumar HS, Karunasagar I. Seasonal variation in abundance of total and pathogenic Vibrio parahaemolyticus bacteria in oysters along the southwest coast of India. Appl Environ Microbiol. 2005;71(7):3575-80.

64. Li R, Chiou J, Chan EW-C, Chen S. A novel PCR-based approach for accurate identification of Vibrio parahaemolyticus. Frontiers in microbiology. 2016;7:44.

65. U.S. Food and Drug Administration, National Shellfish Sanitation Program: Guide for the control of molluscan shellfish (2017).

66. Yang Y, Xie J, Li H, Tan S, Chen Y, Yu H. Prevalence, antibiotic susceptibility and diversity of Vibrio parahaemolyticus isolates in seafood from South China. Frontiers in Microbiology. 2017;8:2566.

67. Rojas MVR, Matté MH, Dropa M, Silva MLD, Matté GR. Characterization of Vibrio parahaemolyticus isolated from oysters and mussels in São Paulo, Brazil. Revista do Instituto de Medicina Tropical de São Paulo. 2011;53(4):201-5.

68. Wagley S, Koofhethile K, Wing JB, Rangdale R. Comparison of V. parahaemolyticus isolated from seafoods and cases of gastrointestinal disease in the UK. Int J Environ Health Res. 2008;18(4):283-93.

69. Klein SL, Lovell CR. The hot oyster: levels of virulent Vibrio parahaemolyticus strains in individual oysters. FEMS Microbiol Ecol. 2017;93(2).

70. Williams TC, Froelich BA, Phippen B, Fowler P, Noble RT, Oliver JD. Different abundance and correlational patterns exist between total and presumed pathogenic Vibrio vulnificus and V. parahaemolyticus in shellfish and waters along the North Carolina coast. FEMS Microbiol Ecol. 2017;93(6):fix071.

Fig1.tif

Click here to download Figure Fig1.tif

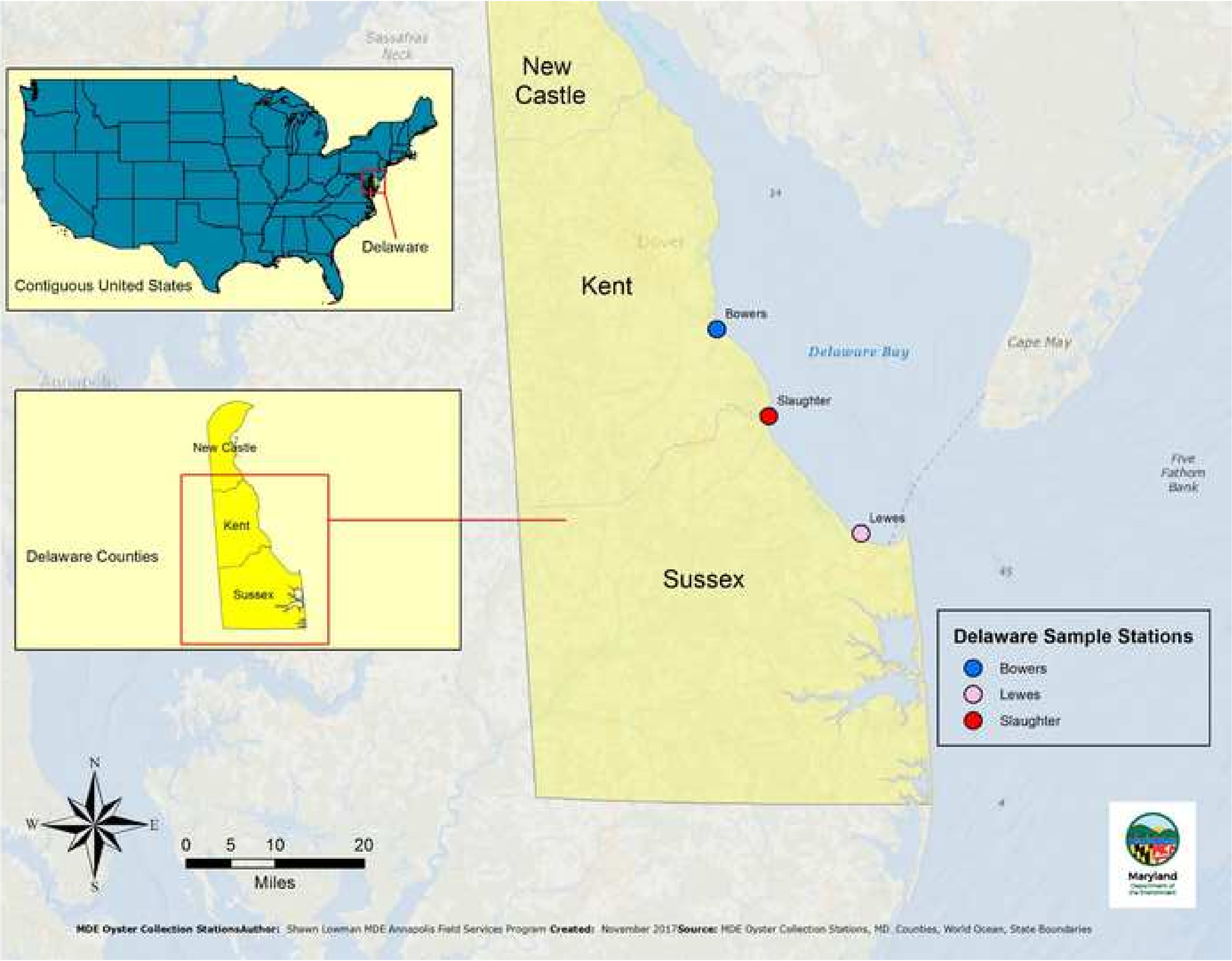

Fig1.tif3.tif

Click here to download Figure Fig2.tif

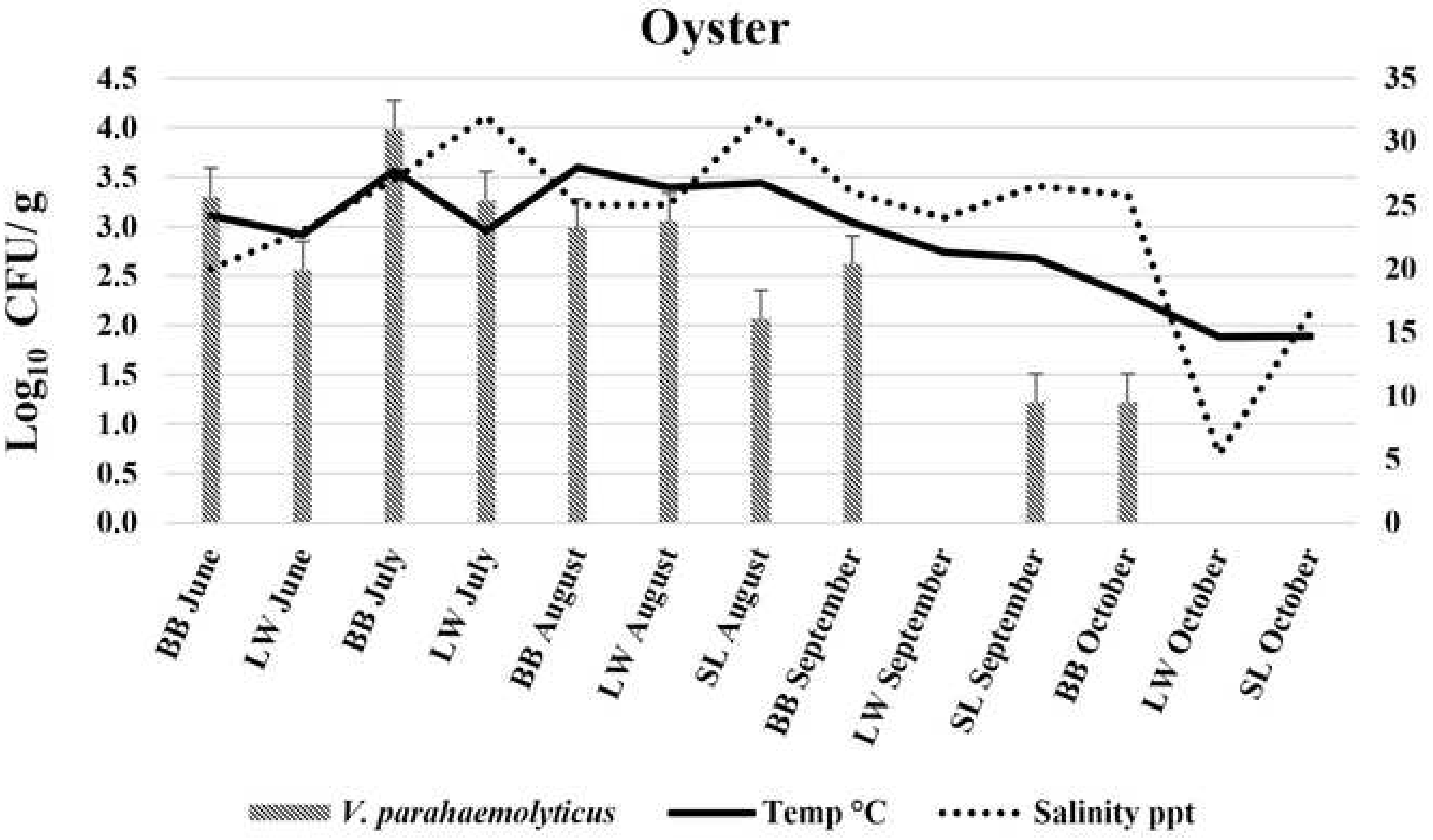

Pasted Layer

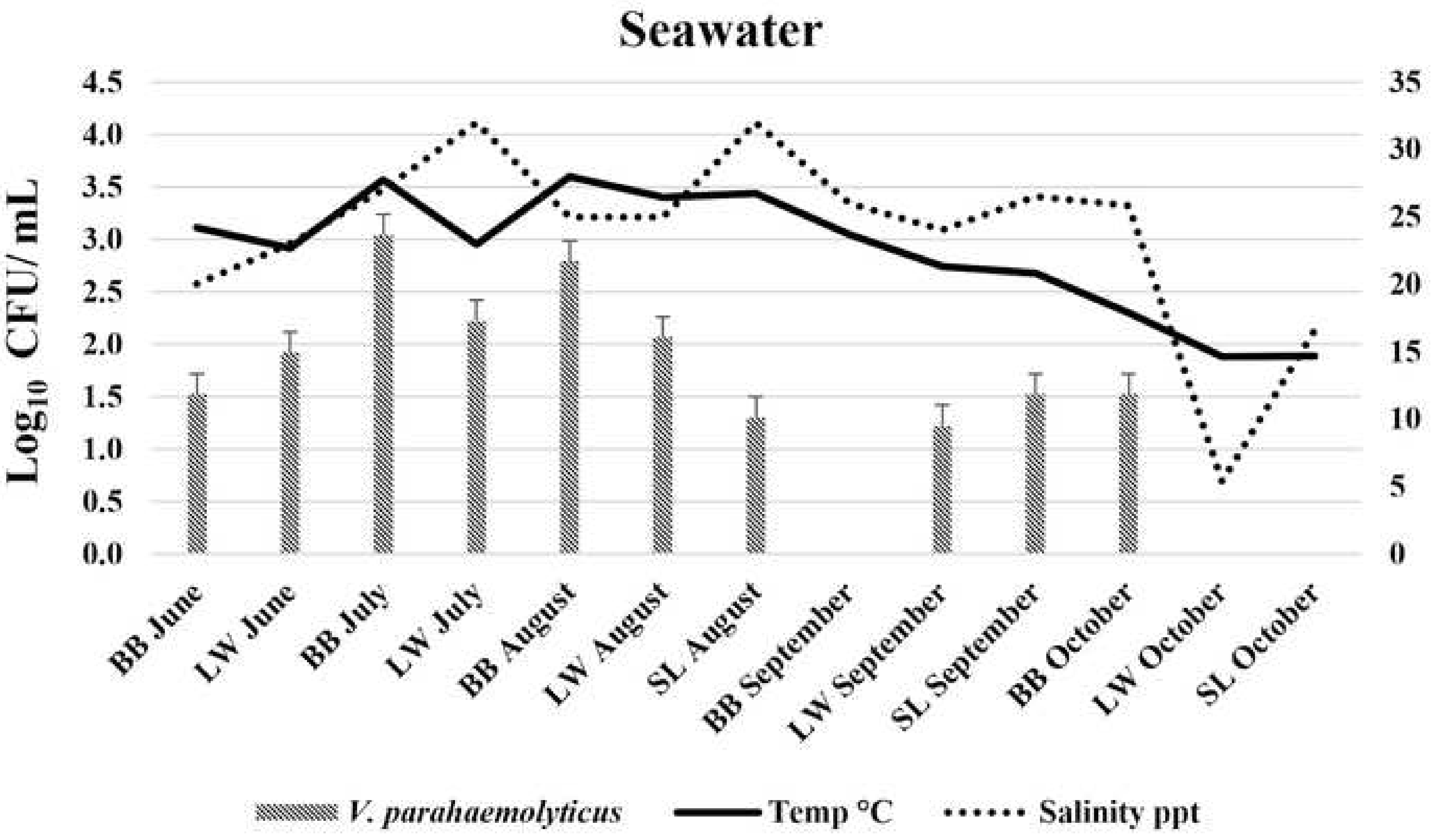

Pasted Layer

Click here to download Figure Fig4.tif

Fig 4.tiff

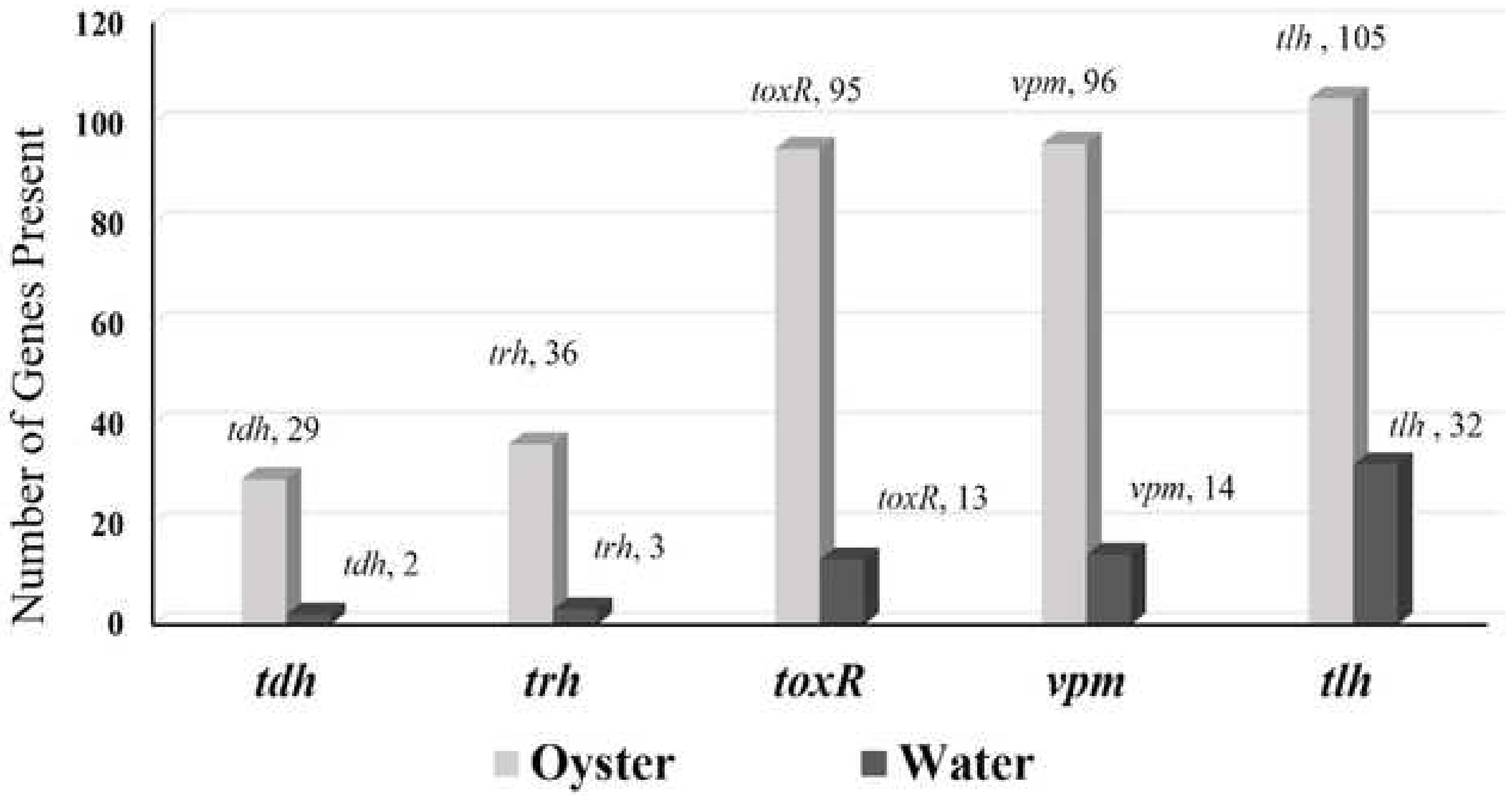

Click here to download Figure Fig5.tif

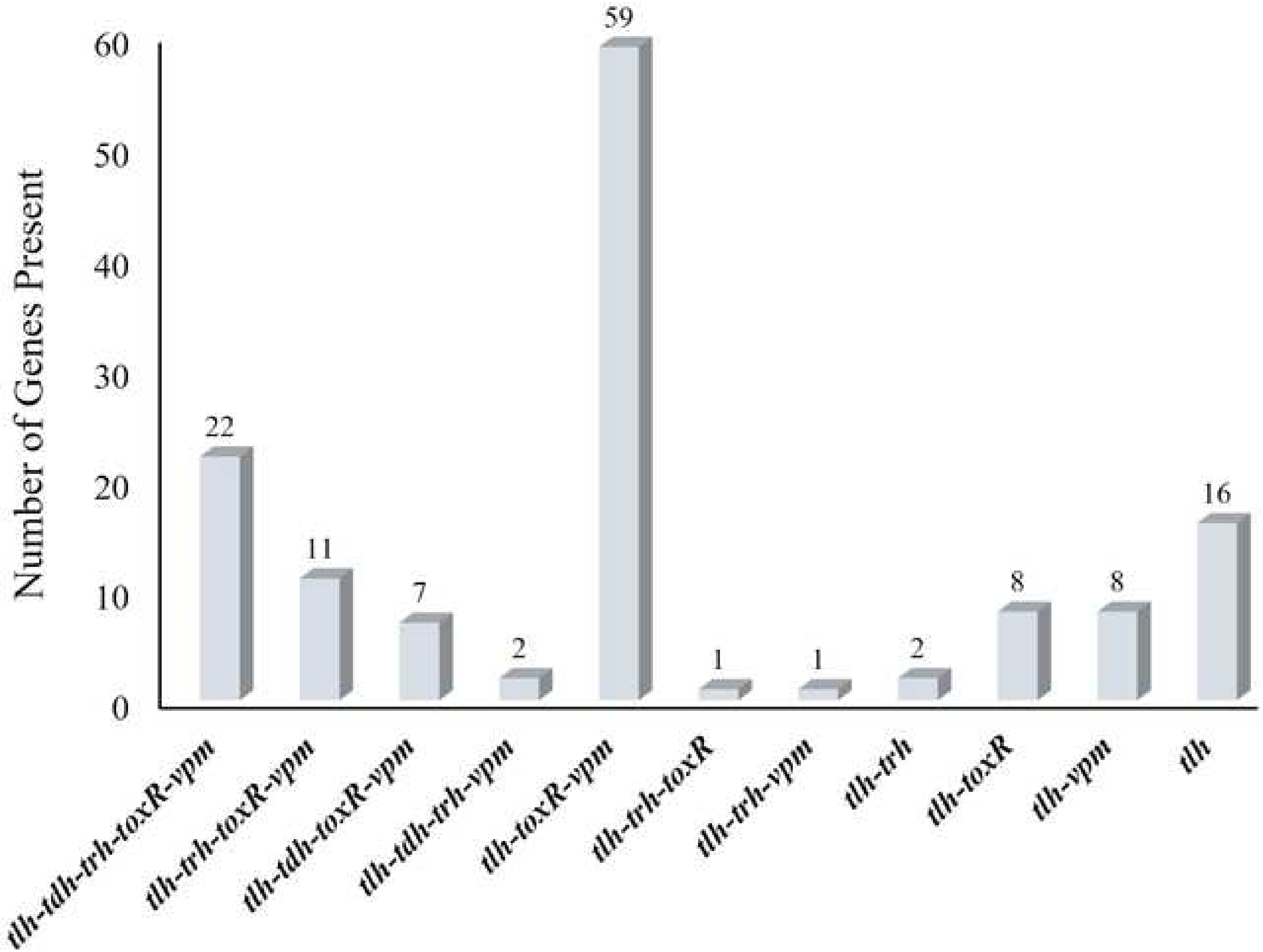

 Click here to download Figure Fig6.tif

Pasted Layer

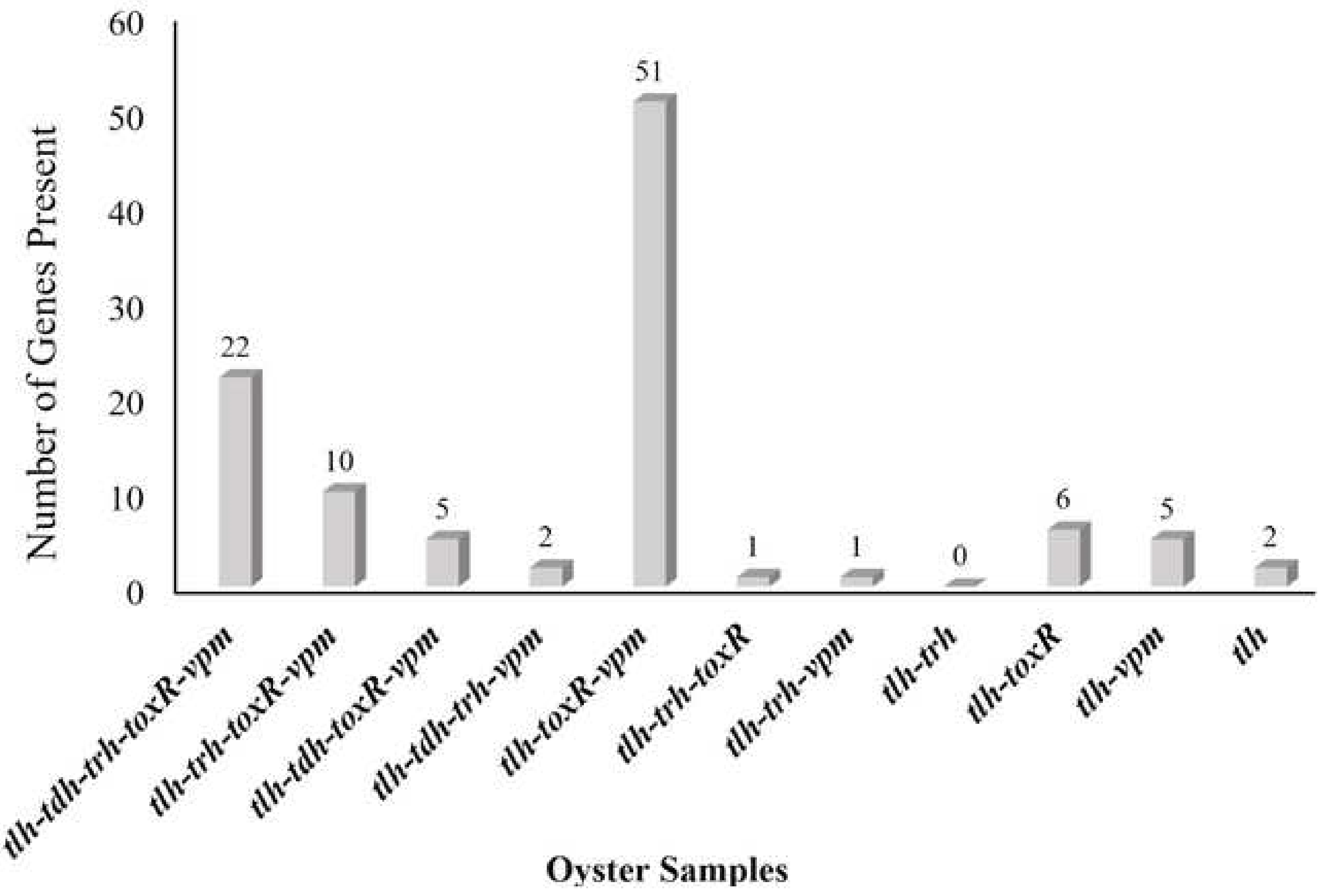

Fig7.tif Click here to download Figure Fig7.tif

Pasted Layer

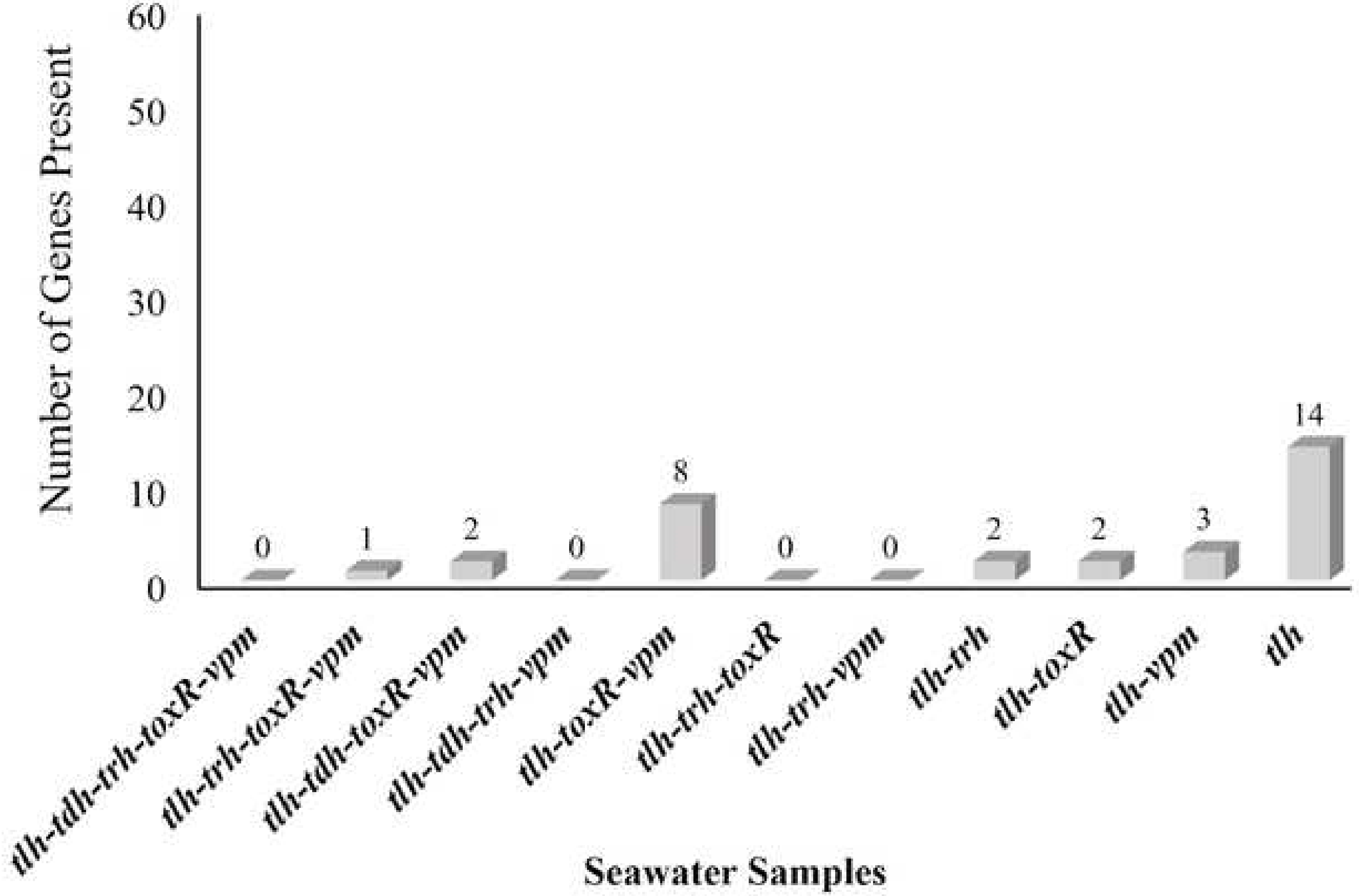

Supplement: Attachment — Submitted filename: PONE-D-20-19028_reviewer_v1.pdf [file pone.0242229.s003.pdf]
