# Supplementary material for: Assessment of Vibrio parahaemolyticus levels in oysters (Crassostrea virginica) and seawater in Delaware Bay in relation to environmental conditions and the prevalence of molecular markers to identify pathogenic Vibrio parahaemolyticus strains
Source: PLoS One. 2020 Dec 3;15(12):e0242229. doi: 10.1371/journal.pone.0242229 (PMC7714158; doi:10.1371/journal.pone.0242229)
Supplement: Attachment — Submitted filename: review_291020.docx [file pone.0242229.s005.docx]

Page 2

L60

*V. parahaemolyticus* is one of the key causes of gastroenteritis should be one of the key …..

L70

*Vibrionaceae*

Pg6 L122-3

Direct plating on CHROMagar Vibrio was used since it is a well-established method, less tedious, and sufficient for the detection and isolation of V. parahaemolyticus [49-51]

Less tedious than what?

What does “sufficient for the detection and isolation” mean?

Pg 6 L143 too many “and” in sentence.

Racine, WI, USA), sub-divided into three groups for biological triplicates (A, B, and C), and placed in an insulated cooler….

Pg7 L157-8

Oysters tissues and liquors from each replicate were placed into a sterilized blender jar (Waring Commercial, 7010S) and blended for 90 sec at high speed.

Pg8 L 169-170

…..plate with a countable range of 20 to 200 colonies was selected to calculate the number of colony forming units (CFU) of the presumptive *V. parahaemolyticus*

173 CM0129) supplemented with 1% NaCl, and incubated with shaking (175 rpm) overnight

L186

186 loading buffer (Taq Buffer; PROMEGA, USA), - do you mean Taq Polymerase buffer?

L189

189 conditions for tlh, tdh, trh, toxR, and vpm genes are shown in (Table 1),

Pg9

L190-192

One μL of nuclease free water was used for the no template control and1 μL of *V. parahaemolyticus* SPRC 10290 cell lysate was used as a positive control.

Pg10

L201-203

For statistical analysis, the CFU values of presumptive V. parahaemolyticus were log10 transformed to normalize the data, and the significance level (P-value) of 0.05 was used.

L212

Physico-chemical water quality parameters (Table 2) showed that water temperatures

Pg12

L242

versa (Table 3). V. parahaemolyticus concentrations from seawater samples were notably lower than oyster samples (Table 3),

Pg13

L253

However, *V. parahaemolyticus concentrations* in the oyster and seawater samples at site BB were 1.7x10 and 3.3x10 CFU/g or mL, respectively.

Pg14

L272

Spearman’s rank correlation analysis showed that water temperature is positively

L275 Salinity had no significant correlation with total V. parahaemolyticus

L281 correlation was found between dissolved oxygen and/or turbidity

L 282 *parahaemolyticus* (Table 4). This result is in contrast to what has been reported from studies conducted in the Chesapeake Bay [57, 58]. Both pH and chlorophyll a did not significantly

Page 15

L282 *parahaemolyticus* (Table 4). This result is in contrast to what has been reported from studies conducted in the Chesapeake Bay [57, 58]. Both pH and chlorophyll a did not significantly

L292-298 A total of 165 presumptive *V. parahaemolyticus* isolates (mauve colored on CHROM agar) were further examined for the presence of the species-specific gene (tlh), and 137 (83%) were confirmed to be *V. parahaemolyticus* (Table 5). Previous investigation revealed that primers targeting the tlh, toxR, and vpm genes were (100%) specific for *V. parahaemolyticus* strains [16]. The lower occurrence of vpm (66.7%) and toxR (65.5%) genes compared to tlh (83%) gene in this study (Table 6 and Fig 4), suggests that tlh gene may occasionally produce false positive results, a ….

L304 *V. parahaemolyticus* (tlh+) were observed among oysters (90.5%) compared to seawater

Pg18

L326 The low occurrence of tdh and trh agrees with the low level of these genes among environmental and food isolates reported in the literature [58, 75-77].

L340 isolates. *V. parahaemolyticus* (tlh+) as illustrated in Table 6 were not proportional to the potential pathogenic *V. parahaemolyticus*,

Pg19

L353 This study also revealed the relatedness potential of tdh occurrence with vpm, as Figs 5, 6, and 7 demonstrated that whenever tdh was present, vpm was also present but not vice versa.
